# Early Recognition of H-Type Tracheoesophageal Fistula

**Published:** 2012-03-01

**Authors:** Muhammad Riazulhaq, Elbagir Elhassan

**Affiliations:** Department of Pediatric Surgery King Faisal Hospital, Taif, KSA

**Keywords:** Tracheoesophageal fistula, H-Type, Esophagus, Atresia

## Abstract

Tracheoesophageal fistula (TEF) without associated esophageal atresia (EA) is a rare congenital anomaly. Diagnosis in neonatal period is usually not made and most of the patients are treated as cases of pneumonia. A case of H-type of tracheoesophageal fistula, diagnosed within 24 hours of delivery based upon choking and cyanosis on first trial of feed, is being reported. Diagnosis was confirmed with contrast esophagram. Through cervical approach fistula was repaired and baby had uneventful post operative outcome.

## INTRODUCTION

H-type TEF accounts for 4-5% of all congenital tracheoesophageal malformations. The clinical features are variable; common being the recurrent respiratory symptoms, aspiration during feeding with cyanosis, and abdominal distension. The early diagnosis of this disorder is difficult and some cases may remain undiagnosed until late in infancy or childhood. The first surgical repair of such a defect was reported by Imperatori in 1939 [1-3]. We are reporting a case of H-type TEF that was diagnosed within 24 hours of birth. 

## CASE REPORT

A term male baby weighing 2.6 kg, born through normal vaginal delivery with good APGAR scores was kept in nursery for observation. As a routine, nasogastric tube was passed without any difficulty. On trial of first feed baby developed choking and cyanosis. The systemic examination was essentially unremarkable. With high suspicion of H-type TEF, a tube esophagram was performed which showed contrast flowing into tracheobronchial tree through a fistula between trachea and esophagus at the level of T1 (Fig.1,2).

**Figure:**
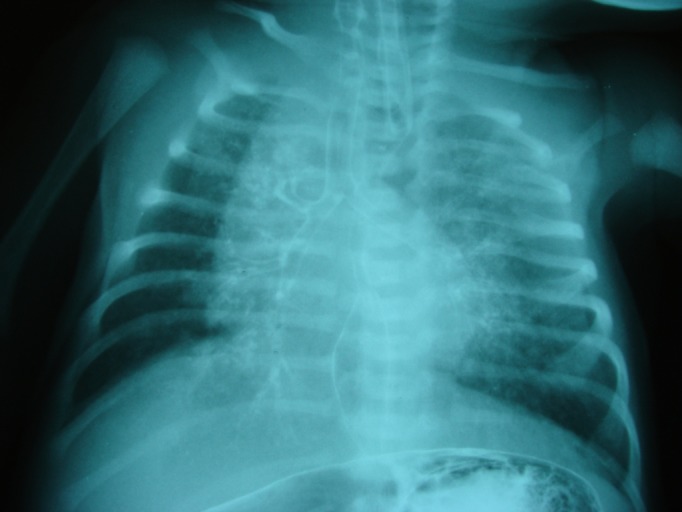
Figure 1: Tube esophagram showing a contrast within the tracheobronchial tree suggestive of TEF.

**Figure:**
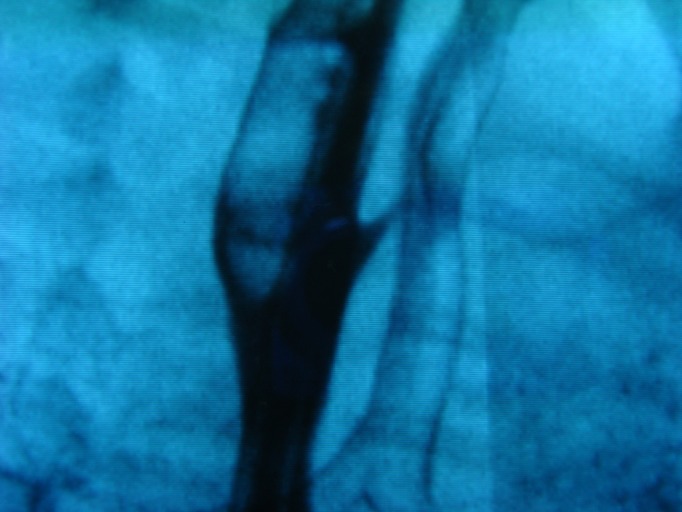
Figure 2: Negative macro image of esophagram delineating fistula between trachea and esophagus.

Oral feed was withheld and baby placed in semi upright position. Intravenous fluids and antibiotics were started. Complete blood count, coagulation profiles and blood chemistry were in normal range. Ultrasound abdomen was unremarkable while echocardiogram showed a small patent ductus arteriosus (PDA). Patient was operated through right low transcervical approach at 36-hour age. Thyroid and cervical trachea were exposed; division of thyroid veins, upward displacement of right lobe of thyroid and lateral displacement of trachea exposed the fistula. Recurrent laryngeal nerve was identified and preserved. Fistula was divided and repaired. After surgery patient was kept on elective ventilation for 72 hours. Nasogastric feed was started on 4th post operative day. Contrast esophagram showed no leakage. Patient was discharged on 10th day of operation after establishing oral feeding.

## DISCUSSION

Most infants with EA with TEF have proximal atresia with distal TEF. They are easily diagnosed soon after birth as to apparent clinical features, nevertheless H-type TEF are not diagnose early because esophagus is patent. Many diagnostic methods have been advocated for the diagnosis of H-type fistula. Esophagram is usually a reliable method to identify congenital H-type tracheoesophageal fistula, though often difficult, requiring multiple attempts before the defect is confirmed. Furthermore, contrast-enhanced studies have the potential risk of aspiration pneumonia and pulmonary injury and should be performed with adequate neonatal emergency resuscitation at hand. Endoscopic methods like bronchoscopy and esophagoscopy have the advantage of being diagnostic allowing placement of a catheter across the fistula to assist in its localization during surgery [2-4]. H-type TEF is associated with other malformations in about 30% of cases, including VACTERL/VATER, CHARGE syndrome, Goldenhar’s syndrome, esophageal stenosis, and syndactyly [5]. The index case has none of these associations.

Different surgical approaches have been described for this anomaly. For proximally located fistula the approach of choice is cervicotomy and in cases of distal fistula thoracotomy is usually preferred. Biechlin et al reported a series of 8 cases of H-type TEF, all were repaired through right cervicotomy. An alternative thoracoscopic approach in a newborn has recently been reported by Allal et al. Surgery consists of ligation and division of the fistula and repair of the tracheal and esophageal walls. Brookes et al reported seven patients of H-type TEF and one patient with a missed proximal H-type fistula associated with esophageal atresia. They presented with coughing while feeding, recurrent pneumonia, and episodic cyanosis. A delay in diagnosis was seen in 4 patients and ranged from 2.5 months to 5.9 years. In all patients, the diagnosis was made on esophagram. The level of the fistulae was between C5 and T3, and all were successfully repaired via a right cervical approach [6-9].

In present case cervical approach was chosen with preservation of recurrent laryngeal nerve. The outcome in present case was satisfactory as baby discharged home on 10th POD in stable condition. A high index of suspicion in cases of cyanosis and choking on first feed and recurrent respiratory symptoms even when esophagus is patent, indicate H-type TEF until proved otherwise. Such patients must be thoroughly investigated to demonstrate the anomaly. 

## Footnotes

**Source of Support:** Nil

**Conflict of Interest:** None declared
